# Efficacy of enhanced *Escherichia coli* phytase on growth performance, bone quality, nutrient digestibility, and metabolism in nursery pigs fed corn-soybean meal diet low in calcium and digestible phosphorous

**DOI:** 10.1093/tas/txac020

**Published:** 2022-03-02

**Authors:** Elijah G Kiarie, Xuerong Song, Junhyung Lee, Cuilan Zhu

**Affiliations:** 1 Department of Animal Biosciences, University of Guelph, Guelph, ON, Canada; 2 Wuhan Sunhy Biology Co. Ltd., Wuhan, P.R. China

**Keywords:** digestibility, metabolism, phytate phosphorous, phytase, pig

## Abstract

Efficacy of *Escherichia coli* phytase (ASP) was evaluated in nursery pigs fed low Ca and digestible P corn and soybean meal diet. Piglets were weaned on day 21, fed a common commercial starter diet for 7 d, and assigned to pens (4 pigs/pen: 2 ♀ and 2 ♂) based on day 7 BW. Positive control (PC) and negative (NC) diets were formulated with similar energy and nutrients with exception of total Ca, total P, and digestible P concentrations being 79%, 67%, and 55% that of PC diet, respectively. Two other diets were formulated by adding ASP in NC at 500 and 1,000 FTU/kg. All diets had 0.2% TiO_2_ indigestible marker. The diets were allocated to pens to give 6 replicates per diet and fed for 42 d. Feed intake and body weight were monitored at 14-d intervals. On day 42, 1 pig/pen was bled and euthanized to access blood and tissue samples. Analyzed total Ca and P in NC diet was 71% and 69% of concentration in PC diet. Recovery of phytase in pelleted diets was 66.2% and 73.5% for NC+500 FTU/kg and NC+1,000 FTU/kg diets, respectively. Between days 15 and 42, pigs fed NC diet grew slower and ate less feed than pigs fed the other diets. Overall (days 0–42), phytase in NC increased (*P* ≤ 0.05) ADG linearly and quadratically. On day 42, pigs fed PC, NC+500 FTU/kg, and NC+1,000 FTU/kg were +6.1, +5.9, and +7.1 kg heavier (*P* < 0.05) than pigs fed NC, respectively. Pigs fed PC and NC plus phytase exhibited higher (*P* = 0.003) G:F relative to NC pigs between days 15 and 28. Pigs fed NC diet had lower (*P* < 0.001) plasma P concentration, apparent total tract digestibility (ATTD) of Ca and P, and metacarpal and metatarsal bone attributes than pigs fed any other diets. Supplementation of phytase in NC linearly increased (*P* < 0.05) plasma P concentration, ATTD of Ca and P, and bone attributes. Specifically, phytase increased (*P* ≤ 0.025) dry weight, length, and ash weight in metacarpals and metatarsals. In conclusion, low total Ca and digestible P diet depressed growth and P utilization in piglets. Supplemental phytase improved performance in pigs fed NC linked to enhanced nutrients uptake and metabolism commensurate to pigs fed adequate total Ca and digestible P from inorganic source.

## INTRODUCTION

Based on well-documented economic and environmental benefits, application of microbial phytase is almost ubiquitous supplement in swine diets (NRC, 2012; Selle et al., 2012; Lei et al., 2013; Markets and Markets, 2020). The majority (~65%) of the P in feedstuffs of plant origin is bound in mixed salts of phytic acids and is unavailable to the animal without enzymatic dephosphorylation (Kiarie and Nyachoti, 2010). Digestibility of P in typical swine diets ranges from 40% to 50% and data indicate that it could be improved up to 60% to 80% depending on phytase dose (Lei et al., 2013). The increase in feed P digestibility, concurrent with the decrease in fecal P concentration, by supplemental dietary phytase can be of tremendous environmental significance as each pig excretes 1.23 kg of P in its full life cycle (Lei et al., 2013). However, in practical swine nutrition, the magnitude of phytase efficacy on the growth performance and P utilization responses is affected by factors ranging from the dietary concentration of phytate and endogenous phytase, level of non-phytate P, Ca, dose and source of the supplemental phytase, and the Ca: P ratio among others factors (Selle et al., 2012; Dersjant-Li et al., 2015; Kiarie et al., 2016; Tsai et al., 2017; Zouaoui et al., 2018).

Innovations for the next generation of phytases are targeting development of molecules showing high tolerance to feed manufacturing processes, high activity at low pH, and efficient degradation of phytate in proximal gastrointestinal tract (Adedokun et al., 2015; Dersjant-Li et al., 2015; Kiarie et al., 2015; Kiarie and Mills, 2019). Moreover, effective and economically viable phytase dose is subject of much scrutiny, more so as the price of inorganic P sources has increased considerably (Markets and Markets, 2020). In a comprehensive review, Dersjant-Li et al. (2015) reported the standard 500 phytase units (FTU)/kg of feed degraded less than 50% of phytate above control in growing pigs, suggesting that higher doses may be more effective in releasing more P. Therefore, we evaluated efficacy of a thermotolerant *Escherichia coli* phytase (ASP) with increased phytate degrading capacity in nursery pigs fed low Ca and digestible P corn-soybean meal diet. The response criteria were growth performance, plasma biochemical profile, minerals digestibility, and bone attributes.

## MATERIALS AND METHODS

Animal care and use protocols were approved by the University of Guelph Animal Care and Use Committee and pigs were cared for in accordance with the Canadian Council on Animal Care guidelines (CCAC, 2009).

### Pigs and Housing

A total of 96 (Yorkshire∗Landrace ♀ x Duroc ♂) 21-d-old weaned pigs (72 barrows and 72 gilts, 6.84 ± 0.49 kg BW) were procured from the University of Guelph’s Arkell Swine Research Station (Guelph, ON, Canada). The piglets were fed a common Arkell swine commercial starter diet for 1 wk after weaning to allow adaptation to the nursery room and solid feed and transitioned to experimental diets at the beginning of second week after weaning. The phytase free starter was corn, and soybean meal based with 2,567 kcal/kg, 23.4%, 1.64%, 0.88%, 0.77% and 0.51% net energy, crude protein, total lysine, total Ca, total P, and available P, respectively (Floradale Feed Mill Ltd., Floradale, ON, Canada). Based on 7-d post-weaning BW, piglets were assigned to pens (4 piglets pen: 2 barrows and 2 gilts) in two environmentally controlled rooms. Each room had 18 pens equipped with a feeder, a nipple type drinker, plastic-covered expanded metal floors, and a wall partitioning between pens that allowed visual contact with pigs in adjacent pens. Room temperature was initially set at 29.5 °C and gradually reduced by 1.5 °C per week.

### Diets and Experimental Procedures

A positive control (PC) was formulated to meet the specifications of nursery pigs (NRC, 2012; Table 1). A second negative control (NC) diet was formulated to have similar nutrients as PC diet with exception that the total Ca, total P, and digestible P concentrations were, respectively, 79%, 67%, and 55 % that of PC diet (Table 1). This was achieved by largely reducing monocalcium phosphate (1.24% vs. 0.26%) in PC vs. NC (Table 1). The total phytate (~0.24%) was similar between PC and NC diet. Two other diets were formulated by adding phytase in NC in place of small amount of cellulose. The intrinsically thermotolerant phytase was from *Escherichia coli* (6-phytase) produced by *Pichia pastoris* expression system (Wuhan Sunhy Biology Co., Ltd., Wuhan, P.R. China). The product was formulated for inclusion at 50 g/ton and as such two formulations with 10,000 and 20,000 µ/g were used in the current study to supply target dosage of 500 and 1,000 FTU/kg of complete feed. All diets had 0.2 % TiO_2_ indigestible marker. Diets were steam pelleted at a temperate range of 65 to 70 °C, and in a die with 410 mm in diameter, effective press width of 114 mm and effective press area of 0.18 m^2^.

**Table 1.** Composition of the positive and negative control diets, as-fed basis

Ingredient name	Positive control	Negative control
Corn	56.9	56.9
Soybean meal 46%	31.8	31.8
Whey permeate	5.95	5.95
Soy oil	0.92	0.99
Monocalcium phosphate	1.24	0.26
Limestone	1.05	1.03
Vitamin and trace mineral premix∗	0.50	0.50
Salt	0.38	0.38
l-Lysine HCL	0.31	0.31
Sodium bicarbonate	0.29	0.29
Titanium dioxide	0.20	0.20
Cellulose	0.20	1.13
dl-Methionine-99%	0.12	0.12
l-Threonine -98%	0.09	0.09
Calculated provisions	100.0	100.0
Net energy, kcal/kg	2,412	2,412
Crude protein, %	20.00	20.00
Standardized ileal digestible Lys, %	1.23	1.23
Standardized ileal digestible Met, %	0.40	0.40
Standardized ileal digestible Met + Cys, %	0.68	0.68
Standardized ileal digestible Thr, %	0.73	0.73
Standardized ileal digestible Trp, %	0.20	0.20
Standardized ileal digestible Val. %	0.81	0.81
Phytate, %	0.24	0.24
Standardized digestible P, %	0.40	0.22
Ca, %	0.80	0.63
Total P, %	0.61	0.41
Ca: total P	1.31	1.54
Ca: Std. digestible P	2.00	2.86
Na, %	0.28	0.28
Zn, mg/kg	123	122
Cu, mg/kg	17.0	16.9

∗Provided per kg of premix: vitamin A, 2,000,000 IU as retinyl acetate; vitamin D_3_, 200,000 IU as cholecalciferol; vitamin E, 8,000 IU as dl-α-tocopherol acetate; vitamin K, 500 mg as menadione; pantothenic acid, 3,000 mg; riboflavin, 1,000 mg; choline, 100,000 mg; folic acid, 400 mg; niacin, 5,000 mg; thiamine, 300 mg; pyridoxine, 300 mg; vitamin B_12_, 5,000 mcg; biotin, 40,000 mcg; Cu, 3,000 mg from CuSO_4_×5H_2_O; Fe, 20,000 mg from FeSO_4_; Mn, 4,000 mg from MnSO_4_; Zn, 21,000 mg from ZnO; Se, 60 mg from Na_2_SeO_3_; and I, 100 mg from KI (DSM Nutritional Products Canada Inc., Ayr, ON, Canada).

The diets were allocated to pens in a completely randomized block (room) design to give 6 replicates per diet. Pigs had free access to experimental diets and water for 42 d, and during this period, feed intake, mortality, and body weight were measured at 14-d intervals for evaluation of growth performance parameters (BW, average daily gain, feed intake, and gain efficiency). Grab fresh fecal samples were collected for 4 d consecutively prior to the end of the trial for determination of apparent total tract digestibility (ATTD) of nutrients. At the end of the trial, one pig per pen (close to the pen average) was bled and euthanized to access tissue samples. Blood samples (10 mL) were collected from orbital sinus bleeding technique (Dove and Alworth, 2015) using a Monoject Standard Hypodermic needle 16 G × 1″ (Covidien; Mansfield, MA) into vacutainer tubes coated with lithium heparin (Becton Dickinson & Co, Franklin Lakes, NJ). The samples were placed on ice, transported to the laboratory, and immediately centrifuged at 2,000 × *g* for 10 min at 4 °C to recover plasma, which was immediately stored at −20 °C until used for analyses. Pig was sedated with a premix of 0.2 mL/kg BW (1 mL containing: Ketamine [50 mg], butorphanol [1 mg], and xylazine [10 mg]) via intramuscular injection followed by intravenous injection of Pentobarbital (Euthansol) at 68 mg/kg BW (Kiarie et al., 2020). The spleen and liver were removed, blotted dry with paper towels, weighed, and discarded. The right feet from the fore- and hind legs were excised for evaluation of metacarpals and metatarsal attributes. Feet were stored at −20 °C in air-tight plastic bags until required for analyses. Jejunal samples (~2 cm middle of small intestine) were be taken, placed in 10% buffered formalin for histomorphology (Kiarie et al., 2018).

### Laboratory Analyses

The plasma samples were analyzed for biochemical profile using a Cobas 6000 C501 Clinical Chemistry Analyzer (Roche Diagnostics, Indianapolis, IN) at the Animal Health Laboratory (University of Guelph, Guelph, ON). The pooled fecal samples were air dried in an oven at 60 °C for 48 h. The feed samples and air-dried excreta samples were finely ground using a coffee grinder (KitchenAid, Mississauga, ON, Canada). All the samples were analyzed for dry matter (DM), N, Ca, P, K, Mg, Na, and Ti. The DM content was determined according to the standard procedures (AOAC International, 2005; method 930.15). Determination of N was carried out by using Leco N analyzer (FP-528; Leco, Saint Joseph, MI) and crude protein calculated by multiplying N values by 6.25. For measuring the mineral content, the samples were dry ashed (ash weight recorded), followed by acid digestion with HCl. Minerals were then analyzed by inductively coupled plasma after appropriate dilution (AOAC, method 985.01). The content of Ti was measured on a UV spectrophotometer according to the method described by Myers et al. (2004). The diets were further analyzed for starch, phytate, and phytase. Crude fat content was determined using an ANKOM XT 20 Extractor (Ankom Technology, Fairport, NY). The content of starch was measured in a commercial laboratory (SGS Canada Inc, Guelph, ON, Canada). The phytase activity was analyzed by AOAC official method (2003): One unit (U) was defined as enzyme needed to liberate 1 μmol inorganic phosphorus per min from 5.0 mmol/L sodium phytate under conditions of pH 5.5 and 37 °C. In the current study, we analyzed the original activity of pure enzyme, the phytase activity in the mash before feed pelleting, as well as the phytase activity in the complete feed after the pelleting process.

Fixed jejunal tissues were embedded in paraffin, sectioned (5 μm), and stained with hematoxylin and eosin at Animal Health Laboratory (University of Guelph, Guelph, ON). In each cross-sectioned tissue, at least 4 to 5 complete villus-crypt structures were examined under a Leica DMR microscope (Leica Microsystems, Wetzlay, Germany), villus height (VH) and crypt depth (CD) were measured using a calibrated micrometer, and the ratio of VH:CD was calculated (Mohammadigheisar et al., 2019). The feet were thawed in fridge overnight and defleshed after autoclaving at 121 °C for 1 min. The feet were then dissected to separate third and fourth metacarpals from fore foot and metatarsals from hind foot. The length and medio-lateral diameter were taken at the mid bone section using a digital calliper with an accuracy of 0.001. The samples were placed in a 70 °C forced-air oven for 4 d with weight recorded before and after drying. They were then fat extracted using hexane for 2 d, dried in a fume hood for 2 d to allow the hexane to evaporate, and ashed at 550 °C in a muffle furnace for 8 h for the determination of ash.

### Calculations and Statistical Analyses

The apparent total tract digestibility (ATTD) of components was calculated according to Kiarie et al. (2018). Data were analyzed using the Mixed model of the GLM procedure of SAS with pen as the experimental unit. The model had diet as a fixed effect and block (room) as a variable effect. Contrast coefficients for supplemental phytase in NC were generated using the interactive matrix language procedure of SAS. An α level of *P* ≤ 0.05 was used as the criterion for statistical significance.

## RESULTS

The analyzed chemical composition of experimental diets is shown in Table 2. The concentration of crude protein, crude fat, and starch in PC and NC diets was comparable. Analyzed concentrations of total Ca and P in NC diet were 71% and 69% of concentrations in PC diet. Although mash diets assayed higher phytase than target, only 66.2% and 73.5% of 500 and 1,000 FTU/kg targets, respectively, were detectable in pelleted diets (Table 2). In the first 14 d of diet exposure, there were no (*P* > 0.05) diet effects on body weight (BW), average daily gain (ADG), and average daily feed intake (ADFI; Table 3). Pigs fed NC diet were lighter (*P* < 0.0001) and grew slower than pigs fed the other diets from day 15 (Table 3). However, between days 29 and 42, there were no differences (*P* > 0.05) on BW and ADG in pigs fed PC and NC diets with phytase. Supplementation of phytase in NC increased (*P* ≤ 0.05) ADG linearly and quadratically between days 15 and 42 and in the overall days 0–42 ADG. At the end of 6 weeks, pigs fed PC, NC+500 FTU/kg, and NC+1,000 FTU/kg were, respectively, +6.1, +5.9, and +7.1 kg heavier (*P*<0.05) than pigs fed NC. Overall (days 0–42), pigs fed NC had decreased (~−20%; *P* = 0.001) ADFI relative to pigs fed PC. Phytase supplementation in NC diet increased (*P* ≤ 0.02) ADFI linearly and quadratically throughout the experimental period. Specifically, in the overall (days 0–42), pigs fed NC diet with 500 and 1,000 FTU/kg consumed 32% and 30% more feed than pigs fed NC diet without phytase. In the first 2 wk, pigs fed PC, or NC plus 1,000 FTU/kg had better (*P* < 0.05) G:F than pigs fed NC pigs, whereas pigs fed NC+500 FTU/kg had intermediate gain efficiency. Between days 15 and 28, pigs fed PC and NC with phytase exhibited higher (*P* = 0.003) G:F relative to NC pigs. Supplemental phytase increased (*P* ≤ 0.01) G:F linearly between days 0 and 28. There were no diet effects (*P* > 0.05) on G:F in between days 29 and 42 and in the overall (days 0–42). Similarly, no (*P*>0.05) effects of diets on liver and spleen weight (Table 3).

**Table 2.** Analyzed chemical composition of experimental diets, as-fed basis

Item	Positive control	Negative control		
Target phytase, FTU/kg	0	0	500	1,000
Moisture, %	10.9	11.3	10.8	10.4
Crude protein, %	20.4	20.4	21.0	20.7
Crude fat, %	2.42	2.43	2.71	2.70
Starch, %	56.7	56.7	57.4	56.1
Ash, %	5.51	5.06	4.89	4.65
Calcium, %	0.75	0.53	0.50	0.56
Total P, %	0.57	0.41	0.39	0.38
Potassium, %	0.98	1.00	0.97	0.99
Magnesium, %	0.18	0.16	0.17	0.16
Sodium, %	0.31	0.31	0.30	0.32
Phytate, %	0.31	0.28	0.27	0.28
Phytase∗, FTU/kg				
Target	0	0	500	1,000
In mash	Undetected	Undetected	1,020	1,360
In pellet	Undetected	Undetected	675	1,000
Pellet/mash ratio, %	–	–	66.2	73.5

∗Provided by Wuhan Sunhy Biology Co. Ltd.

**Table 3.** Growth performance organ weight in nursery pigs fed low calcium and phosphorous corn-soybean meal diet supplemented with *Escherichia coli* phytase

Item	PC	NC			SEM	*P*-value	Phytase response in NC	
							Linear	Quadratic
Target phytase, FTU/kg	0	0	500	1,000				
Body weight, kg								
Day 0 (initial)	7.63	7.73	7.80	7.63	0.09	0.521	–	–
Day 14	12.6	12.4	12.8	12.8	0.28	0.669	0.407	0.658
Day 28	23.0^a^	19.9^b^	22.9^a^	23.1^a^	0.54	0.001	0.009	0.163
Day 42	33.0^a^	26.9^b^	32.8^a^	34.0^a^	0.52	<0.001	<0.001	0.022
ADG, g/d								
Days 0–14	353	333	357	371	14.6	0.345	0.068	0.734
Days 15–28	746^a^	536^b^	719^a^	736^a^	22.8	<0.001	0.001	0.050
Days 29–42	710^b^	497^c^	710^b^	776^a^	20.8	<0.001	<0.001	0.005
Days 0–42	603^a^	455^b^	596^a^	628^a^	11.3	<0.001	<0.001	0.006
ADFI, g/d								
Days 0–14	492	506	520	505	15.0	0.654	0.974	0.517
Days 15–28	933^a^	769^b^	925^a^	918^a^	43.9	0.046	0.054	0.205
Days 29–42	1,103^a^	742^b^	1,209^a^	1,204^a^	52.7	<0.001	<0.001	0.002
Days 0–42	843^a^	671^b^	885^a^	871^a^	33.0	0.001	0.002	0.023
G:F, g/g								
Days 0–14	0.717^a,b^	0.660^c^	0.687^bc^	0.735^a^	0.01	0.008	0.006	0.627
Days 15–28	0.803^a^	0.698^b^	0.781^a^	0.807^a^	0.02	0.003	0.001	0.233
Days 29–42	0.653	0.687	0.593	0.648	0.04	0.459	0.470	0.115
Days 0–42	0.718	0.683	0.676	0.725	0.02	0.291	0.162	0.281
Organ weight, g/kg BW								
Liver	23.5	26.5	24.1	25.9	1.02	0.190	0.718	0.179
Spleen	1.78	2.24	1.82	1.78	0.14	0.054	0.022	0.243

Positive control (PC) and negative (NC) diets had similar energy and nutrient with exception of total Ca, total P, and digestible P concentrations being 79%, 67%, and 55% that of PC diet, respectively.

Within a row, LSmeans assigned different letter scripts differs, *P* < 0.05.

The impact of diets on bone attributes is shown in Table 4. Pigs fed NC diets had lighter and shorter metacarpals and metatarsals relative to pigs fed PC diet. Moreover, piglets fed NC diet exhibited lower ash concentration in the metacarpals (315 vs. 393 mg/g dry weight; −19.8%) and metatarsals (263 vs. 349 mg/g dry weight; −24.6%) than PC pigs. Supplemental phytase in NC diets improved (*P* < 0.05) tested bone attributes. Specifically, the weights of metacarpals and metatarsals of NC+1,000 FTU/kg fed pigs were similar (*P* > 0.05) to PC pigs but heavier (*P* < 0.05) than for NC pigs. Supplemental phytase increased metacarpals and metatarsals dry weight and length linearly (*P* ≤ 0.007) and ash weight linearly (*P* ≤ 0.023) and quadratically (*P* ≤ 0.025).

**Table 4.** Metacarpals and metatarsals attributes in nursery pigs fed low calcium and digestible phosphorous corn-soybean meal diet supplemented with *Escherichia coli* phytase

Item	PC	NC			SEM	*P*-value	Phytase response in NC	
							Linear	Quadratic
Target phytase, FTU/kg	0	0	500	1,000				
Metacarpals∗								
Fresh weight, mg/ kg BW	569	553	487	537	32.5	0.335	0.774	0.251
Dry weight, mg/ kg BW	274^a,b^	244^c^	254^bc^	285^a^	8.59	0.014	0.004	0.309
Length, mm	41.8^a^	39.8^b^	42.7^a^	43.3^a^	0.64	0.007	0.001	0.146
Diameter, mm	13.5	13.1	13.4	13.9	0.23	0.124	0.048	0.601
Ash weight, mg/g dry weight	393^a^	315^b^	390^a^	371^a^	13.9	0.003	0.023	0.025
Metatarsals^†^								
Fresh weight, mg/ kg BW	693	626	612	627	40.6	0.514	0.999	0.823
Dry weight, mg/ kg BW	329^ab^	279^c^	302^bc^	334^a^	10.5	0.004	0.007	0.755
Length, mm	48.7^a^	45.0^b^	47.7^a^	48.9^a^	0.85	0.017	0.002	0.400
Diameter, mm	13.4	13.2	13.1	13.6	0.28	0.642	0.294	0.494
Ash weight, mg/g dry weight	349^a^	263^b^	339^a^	322^a^	12.2	0.002	0.008	0.012

Positive control (PC) and negative (NC) diets had similar energy and nutrient with exception of total Ca, total P, and digestible P concentrations being 79%, 67%, and 55% that of PC diet, respectively.

∗Values are averages of third and fourth.

Within a row, LSmeans assigned different letter scripts differs, *P* < 0.05.

In terms of plasma biochemical constituents evaluated at the end of 6-wk trial, diets had no (*P* > 0.05) effects on concentration of albumin, globulin, urea nitrogen, bilirubin, creatinine, glucose, cholesterol, and haptoglobin (Table 5). The pigs fed NC+1,000 FTU/kg had lower (*P* = 0.039) plasma chloride than pigs fed NC, whereas other pigs were intermediate. Pigs fed NC diet had lower (*P* < 0.001) plasma P concentration than pigs fed any other diet. Specifically, plasma concentration of P was 1.62-, 1.23-, and 1.35-fold higher in PC, NC+500 FTU/kg, and NC+1000 FTU/kg, respectively, than in pigs fed NC diets. Supplemental phytase had a linear reduction (*P* = 0.004) on plasma concentration of chloride in pigs fed NC diet. Phytase increase in plasma P concentration was both linear and quadratic (*P* < 0.001). There were no (*P* > 0.05) diet effects in plasma concentration of calcium, potassium, sodium, and magnesium (Table 4). Pigs fed NC diet had higher (*P* ≤ 0.045) concentration alkaline phosphatase (ALP; 685 vs. 235 U/L), aspartate aminotransferase (AST; 73.5 vs. 46.3 U/L), and γ-glutamyl transferase (GGT; 48.3 vs. 37.8 U/L) than pigs fed PC diet. Supplementation of phytase in NC reduced plasma concentration of ALP (*P* < 0.001; linearly and quadratically), AST (*P* = 0.002; linearly), and GGT (*P* = 0.03; linearly; Table 5).

**Table 5.** Plasma biochemical profile in nursery pigs fed low calcium and digestible phosphorous corn-soybean meal diet supplemented with *Escherichia coli* phytase

Item	PC	NC			SEM	*P*-value	Phytase response in NC	
							Linear	Quadratic
Target phytase, FTU/kg	0	0	500	1,000				
Metabolites								
Total protein, g/L	55.7	53.2	53.3	54.2	1.08	0.366	0.418	0.753
Albumin (A), g/L	45.7	42.8	42.8	44.2	1.07	0.227	0.386	0.614
Globulin (G), g/L	10.0	10.3	10.5	10.0	1.04	0.982	0.835	0.810
A:G ratio	4.68	4.22	4.50	4.84	0.53	0.863	0.458	0.961
Urea nitrogen, mmol/L	5.90	4.45	4.30	5.43	0.45	0.058	0.059	0.144
Haptoglobin, g/L	0.30	0.94	0.58	0.44	0.27	0.557	0.348	0.814
Creatinine, µmol/L	84.0	85.2	79.5	82.5	4.08	0.783	0.643	0.388
Glucose, mmol/L	7.47	7.13	7.13	7.78	0.31	0.419	0.145	0.389
Cholesterol, mmol/L	2.07	1.99	1.92	1.99	0.09	0.682	1.000	0.389
Calcium, mmol/L	2.85	2.81	2.75	2.92	0.54	0.183	0.104	0.077
Chloride, mmol/L	99.2^ab^	100.2^a^	98.5^ab^	97.7^b^	0.57	0.039	0.004	0.517
Potassium (K), mmol/L	9.33	8.42	9.08	8.75	0.31	0.221	0.517	0.269
Sodium (Na), mmol/L	140	138	137	138	1.32	0.361	0.911	0.404
Na: K ratio	15.2	16.5	15.2	16.0	0.61	0.347	0.605	0.206
Magnesium, mmol/L	0.78	0.82	0.77	0.80	0.02	0.524	0.592	0.135
Phosphorous, mmol/L	3.06^a^	1.17^c^	2.61^b^	2.75^ab^	0.12	<0.001	<0.001	<0.001
Enzymes, U/L								
Alkaline phosphatase (ALP)	235^b^	685^a^	213^b^	238^b^	27.4	<0.001	<0.001	<0.001
Aspartate aminotransferase (AST)	46.3^b^	73.5^a^	54.3^ab^	44.8^b^	4.54	0.002	0.002	0.479
Creatine kinase (CK)	7,045	6,869	4,967	4,787	1,292	0.474	0.289	0.607
γ-glutamyl transferase (GGT)	37.8^b^	48.3^a^	42.7^ab^	37.0^b^	3.37	0.045	0.026	0.358

Positive control (PC) and negative (NC) diets had similar energy and nutrient with exception of total Ca, total P, and digestible P concentrations being 79%, 67%, and 55% that of PC diet, respectively.

Within a row, LSmeans assigned different letter scripts differs, *P* < 0.05.

Data for jejunal histomorphology and ATTD of nutrients are shown in Table 6. Pigs fed PC and NC+1,000 FTU/kg diets had taller (*P* = 0.004) villi height than pigs fed NC diet. Diets had no effect (*P* > 0.05) on crypt depth. However, pigs fed PC and NC+1,000 FTU/kg diets had higher VH:CD ratio that pigs fed NC diets. Supplemental phytase linearly increased VH (*P* = 0.006) and VH:CD (*P* = 0.002). The ATTD of DM and CP was similar in pigs fed NC and NC+1,000 FTU/kg but higher (*P* = 0.01) than PC and NC+500 FTU/kg (Table 6). Phytase had quadratic (*P*≤0.048) increase in ATTD of DM and CP. Pigs fed NC diet had lower (*P* > 0.05) ATTD of Ca and P than pigs fed other diets. Phytase supplementation linearly improved (*P* < 0.001) ATTD of Ca and P. Relative to PC fed pigs, phytase improvement of ATTD of Ca was 2% and 22 % for NC+500 and NC+1,000 FTU/kg, respectively. Corresponding values for ATTD of Ca vs. NC were 16% and 39%, respectively. Relative to PC, phytase improvement of ATTD of P was 16% and 29%, respectively, and corresponding values for NC were 38% and 54%, respectively. Pigs fed NC+1,000 FTU/kg had higher (*P* = 0.009) ATTD of K than pigs fed PC diet, whereas ATTD of Na was higher (*P* = 0.012) for pigs fed NC without or with 1,000 FTU/kg than pigs fed PC diet. Diets had no effect (*P* > 0.05) on ATTD of Mg (Table 6).

**Table 6.** Jejunal histomorphology and apparent total tract apparent digestibility (ATTD) of nutrients in nursery pigs fed low calcium and digestible phosphorous corn-soybean meal diet supplemented with *Escherichia coli* phytase

Item	PC	NC			SEM	*P*-value	Phytase response in NC	
							Linear	Quadratic
Target phytase, FTU/kg	0	0	500	1,000				
Jejunal histomorphology								
Villi height (VH), µm	664^a^	609^b^	658^ab^	673^a^	17.8	0.004	0.006	0.419
Crypt depth (CD), µm	132	149	144	140	9.58	0.649	0.481	0.974
VH: CD	5.75^a^	4.59^b^	5.47^ab^	6.20^a^	0.36	0.014	0.002	0.877
ATTD, %								
Dry matter	85.13^b^	88.21^a^	84.57^b^	86.89^a^	0.55	0.001	0.805	0.016
Crude protein	78.95^b^	86.03^a^	79.03^b^	83.33^a^	1.19	0.001	0.667	0.048
Calcium	56.31^b^	49.46^c^	57.34^b^	68.97^a^	1.98	<0.001	<0.001	0.204
Phosphorous	43.19^b^	36.40^c^	50.05^a^	55.91^a^	2.16	<0.001	<0.001	0.457
Potassium	81.70^b^	85.86^ab^	82.74^ab^	86.68^a^	1.06	0.009	0.069	0.100
Magnesium	8.37^b^	15.79^a^	8.07^ab^	6.92^ab^	3.40	0.223	0.285	0.754
Sodium	84.60^b^	89.10^a^	85.63^ab^	89.27^a^	1.09	0.012	0.145	0.127

Positive control (PC) and negative (NC) diets had similar energy and nutrient with exception of total Ca, total P, and digestible P concentrations being 79%, 67%, and 55% that of PC diet, respectively.

Within a row, LSmeans assigned different letter scripts differs, *P* < 0.05.

## DISCUSSION

Reduction of Ca and P in the present study was achieved by reducing monocalcium phosphate to investigate the possibility of restoring growth and mineral utilization by adding phytase. The 18% decline in final BW in pigs fed NC (0.22% digestible P) diet relative to pig fed PC (0.40% digestible P) diet coincided with larger magnitude in bone ash content reduction in metacarpals (−20%) and metatarsals (−25%). Nursery pigs fed −0.19 to −0.17 total P exhibited 9% and 25% depression on body growth and bone mineralization, respectively, relative to pigs fed adequate P diet (Torrallardona and Ader, 2016). Similarly, nursery pigs fed diet with 0.24% digestible P had 15% and 29% lower body weight gain and metacarpal ash content, respectively, relative to control pigs fed 0.42% digestible P (Zeng et al., 2015). Reducing the concentration of P by 50% in nursery piglets reduced growth and bone ash concentration (Lagos et al., 2021). These studies demonstrated essentiality of adequate P nourishment for optimal growth performance and skeletal integrity (NRC, 2012). Moreover, the data extended well documented evidence of the inability of the pig to utilize the P from feedstuffs such as corn and soybean meal. Interesting, the current study suggested that pigs may take more than 2 wk of consumption low Ca and low P diets to observe noticeable effects on growth performance. Similarly, growth performance difference between nursery pigs fed low and adequate Ca and P diets was not detected until after 21 d of exposure (Torrallardona and Ader, 2016). In other published literature, nursery pigs were fed (0.36 vs. 0.22% digestible P) wheat and soybean meal-based diet or (0.35 vs. 0.20% digestible P) corn soybean meal-based diet for a 14-d trial (Dersjant-Li et al., 2017). The data showed that digestible P reduction in wheat-based diets had no effect on growth and gain efficiency but reduced these parameters in corn-based diets. Specifically, low digestible P (0.24% vs. 0.42%) corn and soybean-meal based diet was observed to depress growth after 4 wk of feeding. Such observations suggested considerations for diet types and adequate adaptation period for mineral and phytase studies.

Supplementation of NC diets with phytase significantly improved growth performance and bone mineralization. The efficiency of the phytase in restoring growth performance and bone mineralization to that of the PC fed pigs could be seen at the lowest (500 FTU/kg) inclusion. However, the pig response to phytase dosing was linear and quadratic for some response criteria, suggesting effectiveness of doses between 500 and 1,000 FTU/kg. Supplementation of 500 or 1,000 FTU/kg of *Buttiauxella* phytase in nursery pig diets with 0.20 to 0.22% digestible P and fed for 14 d had no effect on growth performance relative to no phytase supplementation (Dersjant-Li et al., 2017). Supplementation of 500 or 1,000 FTU/kg of *Buttiauxella* phytase in diet with 0.24% digestible P improved growth performance and bone mineralization in nursery pigs relative to no phytase supplementation but had equal and commensurate performance to pigs fed digestible P adequate diet (0.42%; Zeng et al., 2015). Supplementation of phytase doses (125 to 1,000 FTU/kg) from a mixture of bacteria of the species (*Hafnia*, *Yersinia*, and *Buttauxiella*) in low Ca and P diets had linear improvement on nursery growth and bone mineralization relative; however, there was no difference between 500 and 1,000 FTU/kg doses (Torrallardona and Ader, 2016). *Escherichia coli* phytase fed at several doses between 0 and 1,000 FTU/kg in diet with 0.15% digestible P to nursery pigs exhibited linear and quadratic responses on growth and bone mineralization (De Cuyper et al., 2020). Improvements in growth and bone mineralization in the current study and previous studies data can be ascribed to the release of phytate-bound P in the negative control diet. Overall, the growth performance data indicate marginal benefits of 1,000 FTU/kg over 500 FTU/kg.

The plasma biochemical profile helps veterinarians to evaluate health and metabolic status (liver and kidney functioning) through specific grouping of analytes (proteins, enzymes, metabolites, and electrolytes). However, plasma biochemical parameters are influenced by multitude of factors including age, sex, nutritional and health status, breed, season, and stress (Thorn, 2000; Cooper et al., 2014; Abeni et al., 2018). These factors are critical for considerations when interpreting the data. Pigs in the present study were from the same genetic background, healthy, and reared in the same conditions. They were fed isocaloric and iso-nitrogenous, however, relative to PC diet, the NC diet was deficient in digestible P and total Ca. We did not observe difference in the concentration of urea nitrogen, glucose, and cholesterol, indicating balanced supply of energy and amino acids for metabolism. The NC pigs had lower plasma P than PC pigs reflecting insufficient availability at metabolic level as indicated by poor growth and bone mineralization. Supplementation of phytase in NC diets increased plasma P indicating increased release and absorption of phytate bound P in the gut. It is interesting that pigs fed NC diets showed higher concentrations of ALP, AST, and CK and supplemental phytase reduced plasma concentration of these enzymes. Enzymes such as ALP are primarily responsible for bone mineralization, and concentration in blood circulation has been shown to be altered by dietary Ca and P. For example, high plasma ALP concentration has been reported in broiler chickens fed Ca- and P-deficient diets relative to birds fed adequate levels of these minerals (Baradaran et al., 2017; Li et al., 2020). In agreement with the current study, plasma concentration of ALP decreased with supplementation of phytase in broiler diets (Huff et al., 1998).

Phytase has been demonstrated to enhance intestinal barrier function and expression of nutrients transporters in pigs (Lu et al., 2019; Lu et al., 2020). These effects have been associated with mitigation of phytate-induced mucin loss and release of myo-inositol, a precursor for membrane phosphatidylinositol, a component of membrane phospholipids (Onyango et al., 2008; Lu et al., 2019; Lagos et al., 2021). Although these mechanisms were not evaluated in the present study, they could be linked to the improved villi height and villi height to crypt depth in pigs fed phytase. Monogastric foregut is characterized with diminished capability of hydrolysing phytate P (inositol hexaphosphate) in plant feedstuffs due to negligible endogenous phytase activity and low microbial activity (Selle et al., 2012). Phosphorous is primarily absorbed in the form of orthophosphate in the duodenum and jejunum, and thus, hydrolysis of phytate P is critical for enhanced P utilization in plant feedstuffs. Relative to NC pigs, PC pigs had most of digestible P supplied by monocalcium phosphate, but both diets were presented with similar dietary phytate P concentrations (~0.24%). Piglets fed NC without phytase had 16% lower ATTD of P compared to PC fed pigs. Addition of phytase in NC resulted in significant increase in P digestibility in agreement with previous studies (Zeng et al., 2015; Torrallardona and Ader, 2016; Dersjant-Li et al., 2017; Dersjant-Li and Dusel, 2019; De Cuyper et al., 2020). However, although NC+1,000 FTU/kg resulted to the highest ATTD of P relative to NC, this value was still lower than 65%, a maximum ATTD of P indicated by meta-analyses of phytase supplementation in pigs (Rosenfelder-Kuon et al., 2020). The effectiveness of phytase on digestibility of P beyond the levels of P adequate diets (PC) has been demonstrated in other studies. For example, there was no difference in ATTD of P (38.1% vs. 38.3 %) in nursery pigs fed NC (0.12% digestible P) vs. PC (0.26% digestible P; Dersjant-Li and Dusel, 2019). However, supplementation of *Buttiauxella* phytase increased of ATTD of P by 43%, 61%, 79%, and 87% of NC for 250, 500, 1,000, and 2,000 FTU/kg, respectively. Supplemental microbial phytases can also improve digestibility of nutrients other than P, particularly divalent cations such as Ca that can complex phytate in the gut (Selle et al., 2012). In this context, ATTD of Ca was improved by supplemental phytase with NC+1,000 FTU/kg showing higher ATTD of Ca than PC. The efficacy of phytase in releasing complexed minerals has been linked to solubilization of the phytate-cation complexes in the gastrointestinal tract (Dersjant-Li et al., 2015). We did not observe consistent diets differences on ATTD of DM, crude protein, K, Mg, and Na. Similarly, responses of phytase on digestibility of other nutrients other than Ca and P have been very variable (Rosenfelder-Kuon et al., 2020)

These results showed that *Escherichia coli* phytase was effective in improving the utilization of phytate P in corn and soybean meal fed to nursery pigs. Growth rate and bone mineralization in pigs fed low Ca and digestible P diets supplemented with phytase were commensurate to those of pigs fed diets fortified with inorganic P. Although 500 and 1,000 FTU/kg exhibited similar responses, some responses were pronounced at the higher dose in accord with the increased loss of phytate P as phytase activity increased. Indeed, pigs fed 1,000 FTU/kg digested 1.12 times more P than pigs fed 500 FTU/kg.
